# Randomized, Placebo-Controlled, Double-Blind Phase 2 Trial Comparing the Reactogenicity and Immunogenicity of a Single Standard Dose to Those of a High Dose of CVD 103-HgR Live Attenuated Oral Cholera Vaccine, with Shanchol Inactivated Oral Vaccine as an Open-Label Immunologic Comparator

**DOI:** 10.1128/CVI.00265-17

**Published:** 2017-12-05

**Authors:** Samba O. Sow, Milagritos D. Tapia, Wilbur H. Chen, Fadima C. Haidara, Karen L. Kotloff, Marcela F. Pasetti, William C. Blackwelder, Awa Traoré, Boubou Tamboura, Moussa Doumbia, Fatoumata Diallo, Flanon Coulibaly, Uma Onwuchekwa, Mamoudou Kodio, Sharon M. Tennant, Mardi Reymann, Diana F. Lam, Marc Gurwith, Michael Lock, Thomas Yonker, Jonathan Smith, Jakub K. Simon, Myron M. Levine

**Affiliations:** a Centre pour le Développement des Vaccins, Bamako, Mali; b Center for Vaccine Development, University of Maryland School of Medicine, Baltimore, Maryland, USA; c Institute for Global Health, University of Maryland School of Medicine, Baltimore, Maryland, USA; d PaxVax, Inc., Redwood City, California, USA; Food and Drug Administration

**Keywords:** cholera vaccine, immunogenicity, live oral vaccine, Mali, reactive vaccination, single-dose vaccine

## Abstract

Reactive immunization with a single-dose cholera vaccine that could rapidly (within days) protect immunologically naive individuals during virgin soil epidemics, when cholera reaches immunologically naive populations that have not experienced cholera for decades, would facilitate cholera control. One dose of attenuated Vibrio cholerae O1 classical Inaba vaccine CVD 103-HgR (Vaxchora) containing ≥2 × 10^8^ CFU induces vibriocidal antibody seroconversion (a correlate of protection) in >90% of U.S. adults. A previous CVD 103-HgR commercial formulation required ≥2 × 10^9^ CFU to elicit high levels of seroconversion in populations in developing countries. We compared the vibriocidal responses of Malians (individuals 18 to 45 years old) randomized to ingest a single ≥2 × 10^8^-CFU standard dose (*n* = 50) or a ≥2 × 10^9^-CFU high dose (*n* = 50) of PaxVax CVD 103-HgR with buffer or two doses (*n* = 50) of Shanchol inactivated cholera vaccine (the immunologic comparator). To maintain blinding, participants were dosed twice 2 weeks apart; CVD 103-HgR recipients ingested placebo 2 weeks before or after ingesting vaccine. Seroconversion (a ≥4-fold vibriocidal titer rise) between the baseline and 14 days after CVD 103-HgR ingestion and following the first and second doses of Shanchol were the main outcomes measured. By day 14 postvaccination, the rates of seroconversion after ingestion of a single standard dose and a high dose of CVD 103-HgR were 71.7% (33/46 participants) and 83.3% (40/48 participants), respectively. The rate of seroconversion following the first dose of Shanchol, 56.0% (28/50 participants), was significantly lower than that following the high dose of CVD 103-HgR (*P* = 0.003). The vibriocidal geometric mean titer (GMT) of the high dose of CVD 103-HgR exceeded the GMT of the standard dose at day 14 (214 versus 95, *P* = 0.045) and was ∼2-fold higher than the GMT on day 7 and day 14 following the first Shanchol dose (*P* > 0.05). High-dose CVD 103-HgR is recommended for accelerated evaluation in developing countries to assess its efficacy and practicality in field situations. (This study has been registered at ClinicalTrials.gov under registration no. NCT02145377.)

## INTRODUCTION

Cholera, a public health problem that persists among the least privileged populations of many developing countries and can rapidly dehydrate stricken individuals, leading to hypovolemic shock and death without prompt rehydration, is particularly devastating during virgin soil epidemics that reach immunologically naive populations, as seen in Peru in 1991 and Haiti in 2010 (1, 2). In such epidemics, the case fatality rate is often unacceptably high, as the familiarity with cholera and the infrastructure required to treat cholera are typically lacking. Public health authorities have long sought as an adjunct preventive tool a practical formulation of a single-dose cholera vaccine that can rapidly (within days) protect highly susceptible populations harboring no background immunity to Vibrio cholerae O1, to help control epidemic cholera and diminish mortality (3). The oral cholera vaccine Shanchol, which contains a mix of inactivated V. cholerae O1 and O139 strains and is administered as two doses 2 weeks apart, is currently used to control seasonal epidemics of cholera in populations in some developing countries where cholera is endemic.

CVD 103-HgR is a live attenuated Vibrio cholerae serogroup O1, serotype Inaba, classical biotype recombinant strain that harbors deletion of the A (ADP-ribosylating) subunit of classical cholera toxin (CT) but expresses the immunogenic B (binding) subunit, carries an Hg^2+^ resistance marker gene inserted in *hlyA* (thereby inactivating hemolysin A), and expresses the classical toxin-coregulated pilus colonization factor (4–6). A single ≥2 × 10^8^-CFU oral dose of CVD 103-HgR elicits ∼90% seroconversion to serum vibriocidal antibody (7–9), a correlate of protection (9–14).

A CVD 103-HgR formulation containing ∼5 × 10^8^ CFU was originally licensed and commercialized as Orochol and Mutacol by the Swiss Serum and Vaccine Institute (a Berna product) for protection of travelers from industrialized countries (15), while an ∼5 × 10^9^-CFU formulation (Orochol E) was used in developing countries (3). The 1-log-higher dose level was needed to achieve adequate immunogenicity in individuals residing in underprivileged conditions in developing countries (16–19). In 2009, PaxVax, Inc., acquired licensure rights to CVD 103-HgR and is commercializing it as Vaxchora, initially as a cholera vaccine for U.S. travelers (6, 9). A single dose of Vaxchora containing ≥2 × 10^8^ CFU manufactured from the PXVX0200 master cell bank of CVD 103-HgR is highly immunogenic in stimulating serum vibriocidal antibody (a >90% seroconversion rate) and has significantly protected U.S. volunteers against experimental challenge 10 days and 3 months after vaccination (9).

The demonstration in experimental challenge studies in U.S. volunteers that a single ≥2 × 10^8^-CFU dose of Vaxchora provides 91% efficacy against challenge at 10 days after vaccination (9) and documentation by a World Health Organization (WHO) team of the logistical practicality of single-dose use of the previous high-dose formulation of the CVD 103-HgR vaccine (Orochol E) in reactive vaccination to control an explosive cholera epidemic in Micronesia (3) elicited enthusiasm to evaluate a high-dose PaxVax formulation of CVD 103-HgR as a potential future tool for reactive vaccination in explosive virgin soil cholera epidemics in developing countries.

The current study was designed to assess preliminarily in a developing country setting the clinical acceptability and immunogenicity of ≥2 × 10^9^-CFU high-dose versus ≥2 × 10^8^-CFU standard-dose (identical to Vaxchora) PaxVax formulations of CVD 103-HgR and to compare these results with the immune responses following the first and second doses of Shanchol vaccine, which served as an immunologic comparator to give context to the serologic responses to CVD 103-HgR.

## RESULTS

### Participants.

**FIG 1:**
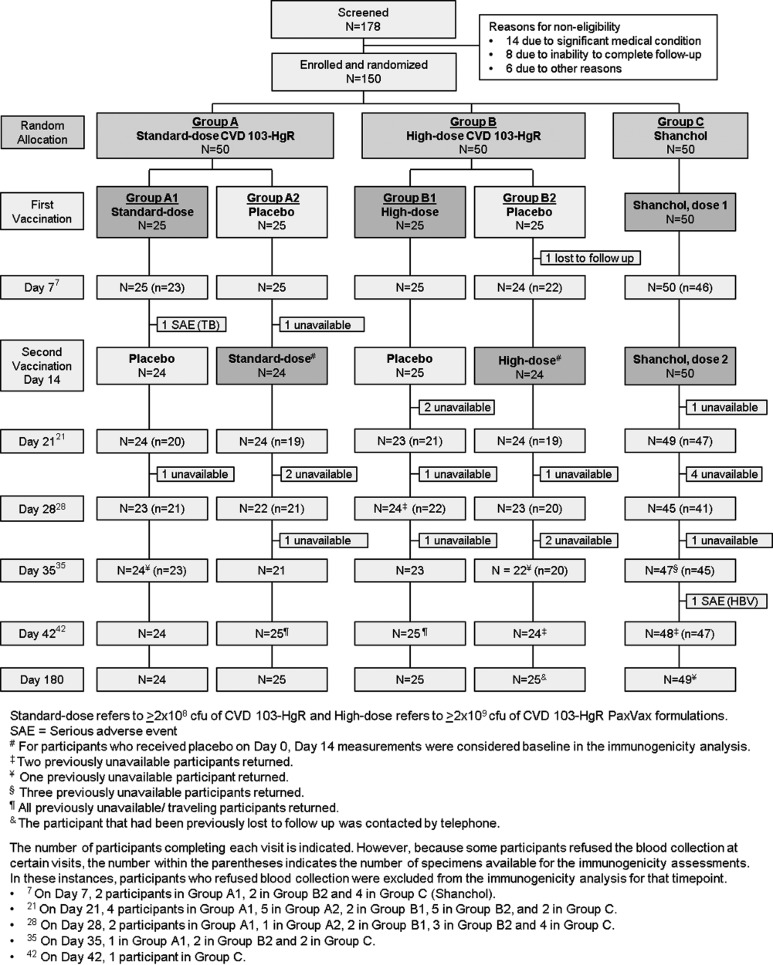
CONSORT (Consolidated Standards of Reporting Trials) diagram.

From 25 August 2014 to 16 September 2014, after informed consent was obtained, 150 adult Malian volunteers were randomly allocated 1:1:1:1:2 to ingest the ≥2 × 10^8^-CFU standard dose of CVD 103-HgR followed 14 days later by ingestion of placebo (group A1, *n* = 25), placebo followed 14 days later by the ≥2 × 10^8^-CFU standard dose of CVD 103-HgR (group A2, *n* = 25), the ≥2 × 10^9^-CFU high dose of CVD 103-HgR followed 14 days later by placebo (group B1, *n* = 25), placebo followed 14 days later by the ≥2 × 10^9^-CFU high dose of CVD 103-HgR (group B2, *n* = 25), or two doses of Shanchol 14 days apart (group C, *n* = 50) (Fig. 1).

Two participants, one each in groups A2 and B2, received placebo on day 0 but did not complete the day 14 visit and did not receive vaccine; one was unavailable due to travel, and the other was lost to follow-up. A third participant, who received ≥2 × 10^8^ CFU of CVD 103-HgR on day 0, was subsequently diagnosed with tuberculosis and excluded from receiving the second scheduled vaccination (placebo). All other study participants received the vaccine (or placebo) to which they were assigned according to the randomization list. There were slightly more females, and group mean ages were 23.9 to 25.0 years (Table 1).

**TABLE 1:** Participant demographics

Characteristic	Values for:		
	Group A (A1 + A2)^*a*^	Group B (B1 + B2)^*b*^	Group C^*c*^
Mean (SD) age (yr)^*d*^	23.9 (6.6)	25.0 (6.6)	24.9 (5.9)
No. (%) of participants in the following age group:			
18–30 yr	43 (86)	40 (80)	42 (84)
31–45 yr	7 (14)	10 (20)	8 (16)
No. (%) of participants of the following gender^*d*^:			
Male	23 (46)	18 (36)	26 (52)
Female	27 (54)	32 (64)	24 (48)

a The participants in group A received CVD 103-HgR at 10^8^ CFU. The 50 participants in this group were randomly allocated to one of two subgroups, with one subgroup of 25 (group A1) receiving the CVD 103-HgR vaccine on day 0 and placebo on day 14 and the other subgroup of 25 (group A2) receiving placebo on day 0 and CVD 103-HgR on day 14.

b The participants in group B received CVD 103-HgR at 10^9^ CFU. The 50 participants in this group were randomly allocated to one of two subgroups, with one subgroup of 25 (group B1) receiving the CVD 103-HgR vaccine on day 0 and placebo on day 14 and the other subgroup of 25 (group B2) receiving placebo on day 0 and CVD 103-HgR on day 14.

c The participants in group B received Shanchol. The 50 participants randomly allocated to this group received one dose of Shanchol on day 0 and the second dose of Shanchol on day 14.

d The differences among the vaccine groups were not statistically significant by age (*P* = 0.66 by ANOVA) or gender (*P* = 0.27 by chi-square test).

### Vaccine safety.

Both CVD 103-HgR dose levels and Shanchol were well tolerated. The only occurrence of diarrhea during the 7 days following ingestion of the study product was in a placebo recipient. Solicited adverse reactions following a dose of CVD 103-HgR at either dose level or following placebo are shown in Table 2. There was no dose-response effect.

**TABLE 2:** Adverse reactions following ingestion of vaccines or placebo

Adverse reaction	No. (%) of participants in:				
	Groups A1^*a*^ and A2^*b*^ (*n* = 49)	Groups B1^*a*^ and B2^*b*^ (*n* = 49)	Placebo^*d*^ (*n* = 99)	Group C^*c*^	
				Dose 1 (*n* = 50)	Dose 2 (*n* = 50)
Any symptom^*e*^	2 (4.1)	2 (4.1)	8 (8.1)	6 (12)	4 (8.0)
Vomiting	0	0	0	0	0
Diarrhea^*f*^	0	0	1 (1.0)	0	0
Fatigue	1 (2.0)	1 (2.0)	2 (2.0)	5 (10)	1 (2.0)
Loss of appetite	0	0	0	3 (6.0)	0
Headache	1 (2.0)	1 (2.0)	5 (5.1)	4 (8.0)	3 (6.0)
Abdominal pain	1 (2.0)	0	1 (1.0)	1 (2.0)	1 (2.0)

a Participants randomly allocated to subgroups A1 and B1 ingested a dose of CVD 103-HgR (∼10^8^ CFU and ∼10^9^ CFU, respectively) on day 0 and a dose of placebo on day 14.

b Participants randomly allocated to subgroups A2 and B2 (∼10^8^ CFU and ∼10^9^ CFU, respectively) ingested a dose of placebo on day 0 and a dose of CVD 103-HgR on day 14.

c Participants randomly allocated to group C ingested their first dose of Shanchol on day 0 and their second dose on day 14.

d Placebo represents the pooled clinical follow-up after the ingestion of placebo by the participants in groups A1, A2, B1, and B2.

e This analysis assessed reactogenicity for the 7 days of active follow-up after each dosing that included multiple household visits following vaccination with CVD 103-HgR and the 7 days of active follow-up that included multiple household visits following receipt of placebo.

f Defined as ≥4 loose stools within a 24-h period.

There were 31 and 20 unsolicited adverse events reported by 19 standard-dose and 18 high-dose CVD 103-HgR recipients, respectively. All reactions were self-limited, resolving within several days. There were no deaths, but two serious adverse events, one for tuberculosis and one for acute hepatitis B virus (HBV) infection, were reported, and both were deemed to be not vaccine related.

### Vaccine immunogenicity. (i) Direct analyses.

Two participants, one each from groups A and B, were excluded from the immunogenicity analyses because they received no active vaccine. In addition, data for 3, 1, and 0 recipients of standard-dose CVD 103-HgR, high-dose CVD 103-HgR, and Shanchol, respectively, were absent from the calculation of day 14 seroconversion rates because of missing values. Seroconversion was observed in 33 of 46 (71.7%) recipients of the standard dose versus 40 of 48 (83.3%) recipients of the high dose of CVD 103-HgR at 14 days after vaccination (*P* = 0.18) (Table 3) (absolute difference, 11.6%; 95% confidence interval [CI], −5.2% to 28.3%). For comparison, the vibriocidal seroconversion rate 14 days after the first dose of Shanchol was 56.0% (28 of 50 recipients; *P* = 0.003 versus high-dose CVD 103-HgR; absolute difference, 27.3%; 95% CI, 9.3% to 43.8%). The seroconversion rate at 28 days after a single high dose of CVD 103-HgR (89.4%) was not significantly different from that at 28 days after initiation of the 2-dose Shanchol regimen (76.1%) (*P* = 0.090) (Table 3). However, at 7 days after the first dose and after the second dose of Shanchol (day 21), the vibriocidal antibody seroconversion rate was also significantly lower than that at 7 and 21 days after the single high dose of CVD 103-HgR (*P* = 0.021 and *P* = 0.018, respectively) (Table 3).

**TABLE 3:** Rates of seroconversion, GMT, and GMFRs following oral vaccination with a single standard dose or high dose of CVD 103-HgR live oral vaccine or two doses (14 days apart) of Shanchol inactivated cholera vaccine^*a*^

Days after vaccination	Seroconversion^*b*^								GMT^*c*^ (95% CI)				GMFR^*d*^ (95% CI)			
	CVD 103-HgR				Shanchol				CVD 103-HgR		Shanchol		CVD 103-HgR		Shanchol	
	10^8^ CFU		10^9^ CFU		After 1st dose		After 2nd dose		10^8^ CFU	10^9^ CFU	After 1st dose	After 2nd dose	10^8^ CFU	10^9^ CFU	After 1st dose	After 2nd dose
	No. of participants who seroconverted/total no. tested (%)	95% CI (%)	No. of participants who seroconverted/total no. tested (%)	95% CI (%)	No. of participants who seroconverted/total no. tested (%)	95% CI (%)	No. of participants who seroconverted/total no. tested (%)	95% CI (%)								
0									28^A^ (21, 39)	28^B^ (21, 38)	47^C^ (32, 69)					
7	22/42 (52.4)	36.4, 68.0	27/44^D^ (61.4)	45.5, 75.6	17/46^E^ (37.0)	23.2, 52.5			99 (57, 171)	199 (106, 375)	102 (65, 160)		3.9 (2.4, 6.3)	7.4^F^ (3.8, 14.2)	2.1^G^ (1.3, 3.4)	
14	33/46 (71.7)	56.5, 84.0	40/48^H^ (83.3)	69.8, 92.5	28/50^I^ (56.0)	41.3, 70.0			95^J^ (56, 161)	214^K^ (117, 394)	128 (75, 218)		3.3^L^ (2.0, 5.5)	7.6^M^ (3.9, 15.1)	2.7^N^ (1.5, 4.8)	
21	36/45 (80.0)	65.4, 90.4	41/46^O^ (89.1)	76.4, 96.4			34/49^P^ (69.4)	54.6, 81.7	73^Q^ (43, 125)	168^R^ (97, 291)		132 (75, 232)	2.8^S^ (1.8, 4.3)	6.2^T^ (3.3, 11.5)		3.0 (1.6, 5.5)
28	36/43 (83.7)	69.3, 93.2	42/47 (89.4)	76.9, 96.5			35/46 (76.1)	61.2, 87.4	75^U^ (46, 123)	167^V^ (111, 253)		135 (81, 226)	2.7^W^ (1.9, 4.0)	5.9^X^ (3.7, 9.5)		3.0^Y^ (1.8, 5.1)

a Seroconversion was considered a ≥4-fold rise in titer over the day 0 baseline titer. The standard dose was ≥2 × 10^8^ CFU, and the high dose was ≥2 × 10^9^ CFU.

b D versus E, *P* = 0.021; H versus I, *P* = 0.003; O versus P, *P* = 0.018.

c A versus C, *P* = 0.045; B versus C, *P* = 0.034; J versus K, *P* = 0.045; Q versus R, *P* = 0.031; U versus V, *P* = 0.014.

d F versus G, *P* = 0.019; L versus M, *P* = 0.052; M versus N, *P* = 0.019; S versus T, *P* = 0.035; W versus X, *P* = 0.012; X versus Y, *P* = 0.057.

There was virtually no difference in the baseline vibriocidal antibody geometric mean titers (GMTs) for participants in the groups receiving the ≥2 × 10^8^-CFU and ≥2 × 10^9^-CFU CVD 103-HgR dose levels, but the baseline vibriocidal GMTs in those groups were significantly lower than the baseline vibriocidal GMT of the Shanchol group (Table 3). In total, 27 of the 150 randomly allocated subjects had moderately elevated baseline reciprocal titers, including 14 with a titer of 160, 9 with a titer of 320, 3 with a titer of 640, and 1 with a titer of 1,280. Fourteen of these 27 individuals were randomly allocated to the Shanchol group, including 7 with a titer of 160, 4 with a titer of 320, and 3 with a titer of 640. The group receiving the standard dose of CVD 103-HgR had 7 individuals with moderately elevated titers (including the subject with a titer of 1,280), while the group receiving the high dose of CVD 103-HgR had 6 subjects with moderately elevated titers.

**FIG 2:**
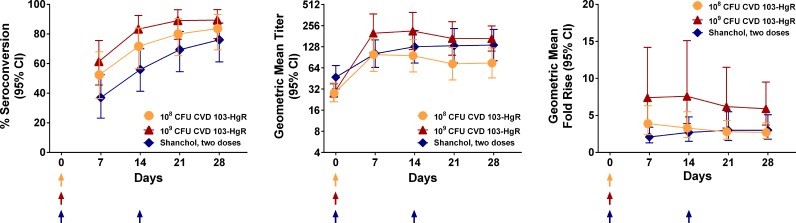
(Left) Percentage of vaccinees in each group who seroconverted by the indicated day of follow-up; (middle) kinetics of the geometric mean titer (GMT) of the serum vibriocidal antibody by the indicated day of follow-up; (right) kinetics of the geometric mean fold rise (GMFR) of the serum vibriocidal antibody by the indicated day of follow-up.

The peak GMT in the recipients of the standard dose of CVD 103-HgR (GMT, 99; 95% CI, 57 to 171) was observed 7 days after vaccination, while the GMT among the recipients of the high dose of CVD 103-HgR (GMT, 214; 95% CI, 117 to 394) occurred on day 14 and was significantly higher than the GMT at day 14 in the standard-dose group (*P* = 0.045). The Shanchol group manifested their peak GMT of 135 (95% CI, 81 to 226) 14 days after ingesting their second dose (28 days after the first dose); this GMT was comparable to the GMT at day 28 for high-dose CVD 103-HgR (Table 3 and Fig. 2).

Because the baseline GMTs of the CVD 103-HgR groups were unexpectedly lower than the baseline GMT of the Shanchol group, despite the random allocation of the participants to groups, a logistic regression analysis that adjusted for the baseline titer was performed. In this analysis, the group receiving the single high dose of CVD 103-HgR still exhibited significantly higher rates of seroconversion by day 14 than the Shanchol recipients 14 days after their first dose of vaccine. Linear regression on the log-transformed vibriocidal titer by use of the log(baseline titer) as a covariate also gave results similar to those obtained by comparison of the unadjusted GMTs. In linear regression on the log(fold rise) with adjustment for log(baseline titer), the geometric mean fold rise (GMFR) over the baseline for high-dose CVD 103-HgR remained higher than the GMFR over the baseline for Shanchol, albeit not significantly (*P* = 0.11). A stratified (Mantel-Haenszel) analysis of seroconversion rates by day 14 in which each observed value of the baseline titer (10, 20, etc.) defined a separate stratum also gave results similar to those in Table 3; the rate of seroconversion for high-dose CVD 103-HgR was higher than that for Shanchol (odds ratio, 4.6; *P* = 0.005).

To perform a survival analysis of seroconversion rates for standard-dose versus high-dose CVD 103-HgR, we compared the seroconversion rates for the entire period through day 28 after vaccination for the primary comparison, standard-dose versus high-dose CVD 103-HgR, using a log-rank test. Though there was a trend during the entire 28-day period toward higher seroconversion rates in the recipients of high-dose CVD 103-HgR, it was not statistically significant (*P* = 0.17).

### (ii) Analysis with imputation for missing values.

The results of the identical analyses summarized in Table 3 but determined using statistical imputation to provide values for the few missing data points (20) are shown in Table S1 in the supplemental material. The results are almost identical to those in Table 3. Table S1 also shows the results of analyses of the effect size comparing the three groups at each time point.

### Vaccine shedding.

The 1,483 stool specimens collected were analyzed for shedding of vaccine organisms; none were positive.

## DISCUSSION

Like other live oral bacterial vaccines, CVD 103-HgR exhibits lower immunogenicity in developing country populations than industrialized country populations (16). To overcome this, a 1-log-higher-CFU dose of the previous commercial formulation (Orochol E) was utilized to achieve high rates of seroconversion with a single dose among serosusceptible individuals of all ages (6, 16–19, 21–23). Expecting that this would also be true with a high-dose PaxVax reformulation of CVD 103-HgR, we conducted a double-blind study of two different CVD 103-HgR dose levels (≥2 × 10^8^ versus ≥2 × 10^9^ CFU) in adults in Mali, West Africa, one of the world's least developed countries. We used the two-dose regimen of Shanchol as an immunologic comparator and assessed vibriocidal antibody titers 7 and 14 days after the first dose and after the second dose of Shanchol.

Extensive evidence shows that the presence of serum vibriocidal antibodies following receipt of a cholera vaccine or after wild-type infection is a correlate of protection against cholera (10, 11, 13, 24). The targets of vibriocidal antibodies are epitopes located on the O1 polysaccharide, which covers vibrios (6, 25). Almost all vibriocidal antibodies can be absorbed with purified O1 polysaccharide, and the initial vibriocidal antibody response resides in the IgM fraction of serum immunoglobulin, but some long-lived IgG antibody also occurs. With repetitive exposure like that which occurs in persons living in areas of endemicity, the proportion of IgG among the vibriocidal antibodies increases. In areas of endemicity, a particular serum vibriocidal antibody titer at the time of presumed exposure correlates with protection (10, 11, 13). More recently, in U.S. volunteers experimentally challenged with wild-type V. cholerae O1 El Tor Inaba following receipt of Vaxchora or placebo, vibriocidal antibody seroconversion, in addition to the peak titer, was a strong correlate of protection (6, 9, 12). Importantly, a precedent to use the noninferiority of vibriocidal antibody seroconversion as the basis for both national regulatory licensure and WHO prequalification of a new inactivated oral cholera vaccine, Euvichol (which has the same composition as Shanchol), has been established (26).

Both dose levels of CVD 103-HgR were well tolerated, and the seroconversion rate among high-dose recipients was higher at all time points postvaccination than among standard-dose recipients, although the differences were not significant. However, the GMT was significantly higher among high-dose CVD 103-HgR recipients than among standard-dose recipients at 14, 21, and 28 days postvaccination. These results are analogous to the findings of studies performed in Peru and Thailand, where the strengths of the standard dose (Orochol) and high dose (Orochol E) of the previous CVD 103-HgR commercial formulations were compared (18, 19).

One limitation of our current study was an overestimation of the likely difference in seroconversion rates between the two CVD 103-HgR dose levels. We had estimated a 29% difference at 14 days after vaccination, based on a Peruvian study of Orochol E versus Orochol (18). Thus, the sample size in the current study was too small and the study was underpowered to find the observed difference in the seroconversion rate (14.4%) to be statistically significant.

Nonliving cholera vaccines, like Shanchol, that require a two-dose regimen have proven effective in dampening seasonal increases in rates of disease in areas where cholera is endemic and where known seasonality and high-risk foci allow targeting of populations (27–29). However, the nonliving vaccines have not performed well when administered as a single dose (27, 30), particularly in immunologically naive persons, such as children <5 years of age in areas of endemicity (who suffer the highest age-specific incidence of cholera). This recognized limitation is most relevant during explosive virgin soil outbreaks, when cholera reaches immunologically naive populations that have not experienced cholera for decades (1, 2). In such instances, the immunologic susceptibility of the population, the suboptimal treatment of cholera due to inexperience, and a lack of infrastructure to transport cholera gravis patients rapidly to treatment centers to receive intravenous and oral rehydration and antibiotics conspire initially to cause unacceptably high case fatality rates and social disruption. Such epidemics can be enormous, as occurred in Peru, Ecuador, and Colombia in 1991 (1) and Haiti in 2010 (2). Other epidemics affecting Pacific Island states have generated fewer cases but have stricken a notable portion of the entire population (3). A single-dose vaccine that confers protection within 8 to 10 days after vaccination would be logistically more practical for reactive mass vaccination to control explosive virgin soil epidemics and diminish mortality than vaccines that require a two-dose regimen with 2 weeks of spacing between doses. There is a similar need for a single-dose, rapidly acting, effective vaccine when cholera reappears unexpectedly after years without disease in a previously affected country, often striking different geographic regions.

Immunogenicity studies with Shanchol in populations in Kolkata, India, and Dhaka, Bangladesh, where cholera is endemic, demonstrated moderate rates of seroconversion 7 to 14 days following the first dose of the two-dose Shanchol immunization regimen (31, 32), generating interest to answer directly the question of whether significant protection might be manifest after a single dose, at least among primed persons in areas of endemicity. Accordingly, an enormous individual-randomized, placebo-controlled trial among 204,700 poor urban residents of Dhaka, Bangladesh, assessed the efficacy of a single dose of Shanchol in preventing cholera in residents of this community, where cholera is hyperendemic. The overall efficacy was modest, 40% (95% CI, 11% to 60%), but statistically significant (30). However, among children 1 to 4 years of age, who are less immunologically primed than older subjects, vaccine efficacy was only 16% (95% CI, −49% to 53%) (30). Similarly, a test-negative case-control study nested within a mass vaccination in Odisha, India, showed that single-dose Shanchol had an effectiveness of 32.5% (95% CI, −318% to 89.5%) overall for individuals of all ages (27).

Herein we report that the high-dose CVD 103-HgR live vaccine formulation gave significantly higher rates of vibriocidal antibody seroconversion and higher GMTs at 7 and 14 days after a single dose than one dose of Shanchol. The significantly stronger immunogenicity of the live vaccine is expected to be accompanied by an enhanced capacity to protect subjects after a single oral dose in developing country settings, and it is important for field studies to investigate this.

Two efficacy studies assessed the ability of Orochol E (10^9^ CFU), the previous commercial high-dose formulation of CVD 103-HgR, to prevent cholera in developing country populations. First, a double-blind, individual-randomized, placebo-controlled trial conducted in North Jakarta, Indonesia (33,696 vaccinees, 33,812 placebo recipients), where cholera is hyperendemic, failed to show significant protection over 4 years of follow-up (33). However, following vaccination there was a precipitous fall in the number of cholera cases in the population overall compared to that in the 4 years before the trial, suggesting that powerful indirect protection diminished the overall risk of cholera in controls as well as vaccinees. Second, during a cholera epidemic on Pohnpei Island, Micronesia, WHO undertook a reactive mass immunization campaign using single-dose Orochol E and calculated 79% vaccine effectiveness (95% CI, 72 to 85%) in preventing cholera under field conditions (3).

In the current study, no CVD 103-HgR vaccinees shed vaccine organisms in their stool, but this was an expected result. From the regulatory authority perspective, an attractive property of CVD 103-HgR identified early in its clinical development is that it elicits high rates of seroconversion to vibriocidal antibody with high GMTs accompanied by only modest excretion, even in North Americans (18 to 25% of subjects) (4, 7, 8). Among vaccinees in developing countries, excretion is minimal or absent, despite ingestion of 1-log more organisms and strong immune responses (16–18, 21, 23, 34). This is believed to be the consequence of the environmental enteropathy present in most children and adults living in poverty in developing countries (16, 35). Indeed, in Peru, where both standard-dose and high-dose CVD 103-HgR were compared, the excretion of CVD 103-HgR was observed in a few percentage of high-socioeconomic-level subjects who received the high dose, while no excretion was seen in low-socioeconomic-level vaccinees who got the high dose (18). Lastly, in a previous trial in Mali, even HIV-positive recipients of the high dose failed to show positive coprocultures, while they exhibited good seroconversion rates (36). This phenomenon of minimal or no shedding in developing country participants has also been described for Peru-15, another single-dose live oral cholera vaccine (37).

Future studies will also include measurements of IgG B memory cells (38, 39) and antitoxin antibodies. Based on the highly encouraging results obtained with high-dose CVD 103-HgR, we will undertake additional clinical trials in sub-Saharan Africa and other less developed regions with a high-dose CVD 103-HgR formulation having nonstringent cold chain requirements and a presentation suitable for practical administration to young children. Evaluations of a single high dose of CVD 103-HgR will aim to gauge its future suitability for use in reactive vaccination campaigns during explosive outbreaks (particularly in cholera-naive populations) (2), for preemptive vaccination to diminish the burden of seasonal endemic cholera, and for protection of high-risk populations in which cholera epidemics have previously posed serious threats (e.g., refugee camps).

## MATERIALS AND METHODS

### Ethics statement.

The clinical protocol (ClinicalTrials.gov registration no. NCT02145377) was approved by the Ethics Committee of the University of Bamako Faculty of Medicine, Pharmacy and Odontostomatology and the Institutional Review Board of the University of Maryland School of Medicine. Informed consent was obtained from healthy adult volunteers 18 to 45 years of age screened for the inclusion and exclusion criteria published at ClinicalTrials.gov (https://clinicaltrials.gov/show/NCT02145377). Participants provided informed consent, documented on a printed form. If the participant was illiterate, consent was obtained after she or he listened, in the presence of a literate witness, to the audiotaped version of the consent form in Bambara.

### Study design and participants.

This single-site trial was conducted at l'Unité Clinique of the Centre pour le Développement des Vaccins, Mali (CVD-Mali), in Bamako, Mali, a country with one intermittent focus of cholera (the Mopti Region). Bamako is free of endemic cholera. Over 3 years there were no isolations of V. cholerae O1 among children <5 years of age with moderate to severe diarrhea in the Global Enteric Multicenter Study (GEMS), whereas repetitive isolations were made from cases in three South Asian sites and Mozambique (40). The trial was conducted from 25 August 2014 to 15 March 2015.

Interested persons presenting to the study site from 25 August 2014 to 16 September 2014 were screened for eligibility. Eligible participants were healthy adults ages 18 to 45 years (inclusive) without a notable medical history who could understand the study and provide informed consent. Women of childbearing potential had negative urine pregnancy tests prevaccination and agreed to employ birth control. Ineligible were persons in direct contact with immunodeficient patients; HIV-positive individuals; persons with unstable medical conditions, a clinically relevant history of immunodeficiency, endocrine disorder, cardiovascular, respiratory, hepatic, renal, or gastrointestinal disease, neurologic illness, a psychiatric disorder requiring hospitalization, current drug or alcohol abuse, a history of abnormal stool pattern or regular laxative use, previous cholera vaccination, cholera illness, or residence of ≥6 months in the Mopti Region or travel there during a cholera outbreak; persons who received or planned to receive another licensed vaccine from 14 days prior to the study vaccination until day 42; persons who received or planned to receive antibiotics within 10 days prior to the study vaccination through to day 28; persons who received systemic chemotherapy within 5 years prior to the study; persons who received or were to receive immunosuppressive therapy, radiation therapy, or high-dose inhaled steroids within 30 days prior to the study vaccination through to day 28; pregnant or breast-feeding women; persons positive by a previous serologic test for hepatitis C or hepatitis B virus; and persons with other conditions that investigators opined might affect participant safety or the ability to evaluate the study product.

### Randomization and masking.

An independent statistician produced a computer-generated list of random allocations in blocks of alternating size of 6 or 12 participants. From 25 August to 16 September 2014, after providing stool and blood samples and in order of arrival into the vaccination room, eligible volunteers were randomly allocated to receive one of three possible treatments: ≥2 × 10^8^ CFU CVD 103-HgR (groups A1 and A2), ≥2 × 10^9^ CFU CVD 103-HgR (groups B1 and B2), or Shanchol (group C). Study products were prepared in a separate room by research pharmacy personnel not blind to the treatment and then brought to the participant for administration. Participants in all three treatment groups received two oral doses spaced 14 days apart. CVD 103-HgR was administered either at day 0 followed by placebo 14 days later (groups A1 and B1) or at day 14 following placebo at day 0 (groups A2 and B2). Thus, there were 4 different randomized allocations of CVD 103-HgR when order was considered; treatment allocations were in the ratio of 1:1:1:1:2. Persons allocated to Shanchol received two oral doses in an open-label fashion. Study personnel who were not blind to the treatment and who administered the study vaccines had no contact with the participants after vaccination and maintained concealment of the product allocation from personnel blind to the product allocation.

### Vaccine.

Formulations of live attenuated vaccine were prepared from a PXVX0200 working cell bank of CVD 103-HgR according to current good manufacturing practices by PaxVax, Inc., Redwood City, CA, and packaged as a lyophilized powder within single-use sachets containing ≥2 × 10^8^ CFU (identical to Vaxchora) or ≥2 × 10^9^ CFU of vaccine organisms and small amounts of stabilizer-filler. An accompanying buffer powder sachet contained 2.5 g NaHCO_3_, 1.6 g ascorbic acid, and 0.2 g lactose. A vaccine dose was prepared by suspending the contents of the vaccine and buffer sachets in a cup containing 100 ml of bottled water. Placebo consisted of 100 ml of bicarbonate buffer. Shanchol, provided by Shantha Biotechnics, a Sanofi Company, Hyderabad, India, was prepared per the instructions in the package insert. After the vial was shaken, the vial contents (1.5 ml) were loaded into a needleless syringe and deposited *per os*.

### Procedures.

Study participants were observed for 30 min after ingesting the study products. The participants were visited at home on days 1, 2, 3, and 7 by clinical team members blind to the product allocation to assess reactogenicity (diarrhea, abdominal pain, vomiting, headache, lethargy, anorexia) for 7 days following each vaccination. Diarrhea was defined as ≥4 loose stools in a 24-h period. Additional clinic visits were completed on days 35 and 42, and a final visit was completed on day 180 (in the clinic or via telephone). The occurrence of adverse events (AE) was recorded through 28 days postvaccination, and information on serious adverse events (SAE) was collected through day 180. A safety monitoring committee reviewed the safety data during the study.

### Bacteriology.

To detect excreted vaccine organisms, stool specimens were inoculated onto thiosulfate-citrate-bile salts-sucrose agar plates (Eiken, Tokyo, Japan) (41). Suspicious colonies were confirmed to be Vibrio cholerae O1 Inaba (putatively the organism in the CVD 103-HgR vaccine) by verifying that the subcultures were oxidase positive and agglutinated by anti-O1 and anti-Inaba antisera.

### Immunology.

Coded serum specimens were tested for Inaba vibriocidal antibody by technicians blind to the product allocation using previously described methods with slight modifications (8, 42). V. cholerae Inaba 89 was plated overnight at 37°C on tryptic soy agar plates containing 5% sheep blood (Becton, Dickinson [BD], NJ). Colonies were transferred to brain heart infusion (BHI; BD) plates and incubated overnight at 37°C. Single colonies were streaked onto duplicate BHI plates and incubated for 4 h at 37°C, after which harvested bacteria were suspended in 0.85% saline to achieve ∼5 × 10^6^ CFU/ml. Serum samples (and controls) were serially diluted in 0.85% saline solution across 96-well plates, whereupon 25 μl of a V. cholerae Inaba suspension containing 5 × 10^6^ CFU/ml and 10% (vol/vol) guinea pig complement was added, and the plates were incubated for 1 h at 37°C. BHI broth (0.15 ml) was added to all wells, and incubation was continued for 3 h at 37°C. A V. cholerae Inaba 89 growth curve established optical density at 620 nm (OD_620_) values corresponding to 0, 25, 50, and 100% viability. The vibriocidal antibody titers of experimental samples were calculated on the basis of the OD_620_ values as the reciprocal of the highest serum dilution that inhibited at least 75% of bacterial growth, determined through the viability curve. Seroconversion was defined as a ≥4-fold rise in the postimmunization titer over the baseline titer.

### Outcomes, statistical analyses, and sample size calculations.

The study compared the rate of seroconversion to serum Inaba vibriocidal antibody following ingestion of one of two different dose levels of CVD 103-HgR with the rates of seroconversion elicited by one or two doses of Shanchol. Seroconversion was defined as a ≥4-fold rise in titer by 14 days after the last dose (compared to that at the baseline). Secondary aims were (i) to compare the mean fold rise in the serum Inaba vibriocidal antibody titer (compared to the baseline titer) and the GMT by 14 days after vaccination with a single ≥2 × 10^8^-CFU standard dose or a ≥2 × 10^9^-CFU high dose of CVD 103-HgR with those achieved with Shanchol, (ii) to plot the kinetics of the serum Inaba vibriocidal antibody response achieved after ingestion of a single oral dose of CVD 103-HgR containing ≥2 × 10^8^ CFU or ≥2 × 10^9^ CFU against those achieved after ingestion of Shanchol, (iii) to assess the fecal shedding of CVD 103-HgR by vaccine recipients, and (iv) to compare the rate of diarrhea (defined as ≥4 loose stools within 24 h) following administration of each vaccine regimen with that following administration of placebo over 7 days of follow-up.

Immunogenicity analyses included all subjects contributing data for both vaccine and placebo treatments. For calculation of the rate of seroconversion to vibriocidal antibody by 14 days after ingestion of a dose of CVD 103-HgR or Shanchol, subjects with missing values at day 7 or 14 were not included unless there was seroconversion based on the nonmissing values. Missing values were handled similarly in the calculation of seroconversion rates at 21 and 28 days after vaccination. Vibriocidal antibody responses were also summarized by GMT and GMFR, along with the corresponding two-sided 95% confidence intervals (CIs), from the baseline to day 14. Two-sided 95% CIs for differences between seroconversion rates were calculated by a likelihood score method. CIs for ratios of GMTs and ratios of GMFRs were calculated by exponentiating the confidence limits for differences between the means of the log-transformed titers and the fold rises, based on *t* distributions. Seroconversion rates were compared by chi-square tests, and GMTs and GMFRs were compared by two-sided *t* tests on log-transformed titers.

Since some participants failed to appear for a follow-up visit, there were missing values because some specimens had not been obtained. In addition to analyses of the data as collected, seroconversion rates, GMTs, and GMFRs were estimated using multiple imputations of missing titer values. We did 100 imputations by fully conditional specification (20), which requires no specification of distributions, using the PROC MI and PROC MIANALYZE procedures in SAS (version 9.4) software (SAS Institute, Cary, NC) for point estimates and effect sizes, two-sided 95% CIs, and pairwise comparisons. CIs on the imputed data were based on *t* distributions, and comparisons were done using *t_i_* tests.

Adjustment for the baseline titer was made by logistic regression on seroconversion (yes/no) or linear regression on the log-transformed titer or the log-transformed fold rise, with the baseline vibriocidal titer or its logarithm being used as a covariate. In addition, pairwise comparisons of seroconversion rates by day 14 were done in a stratified (Mantel-Haenszel) test in which each observed value of the baseline titer (10, 20, etc.) was considered a separate stratum.

Safety results were summarized for the recipients of CVD 103-HgR, Shanchol, and placebo by the percentage of study participants experiencing various reactions by day 7 after vaccination. Safety endpoints were compared using a two-sided Fisher's exact test, with the *P* value being calculated by doubling of the lower of the two one-sided *P* values, to account for unequal sample sizes. We combined the placebo groups in order to maximize the power to find a statistically significant increase in a reaction with any vaccine, ignoring any correlation induced by some participants contributing data for both vaccine and placebo.

Comparisons of age and gender distributions among the groups were done by analysis of variance (ANOVA) and the chi-square test, respectively. All comparisons were done according to original treatment assignment. Statistical significance was indicated by a *P* value (two-sided, where appropriate) of ≤0.05. *P* values were not adjusted for multiplicity.

The true underlying vibriocidal seroconversion rates were anticipated to be ∼72% after a ≥2 × 10^9^-CFU dose and ∼43% after a ≥2 × 10^8^-CFU dose, on the basis of results of a study of Orochol E versus Orochol in Peruvians (18). For these estimated seroconversion rates, 46 subjects per group with analyzable data would provide an ∼81% power to detect a significant difference (two-sided *z* test, alpha = 0.05). We proposed enrolling 50 subjects per group to mitigate against the loss to follow-up of up to 4 subjects per group. If data for all 50 subjects were available for analysis, we would have an ∼86% power to detect a significant difference between groups.

The rate of diarrhea (4 or more watery stools in a 24-h period) in the placebo group was expected to be <2%. The diarrhea rate in recipients of a ≥2 × 10^8^-CFU dose or a ≥2 × 10^9^-CFU dose of CVD 103-HgR was expected to be only slightly higher (∼4%). Should the rate of diarrhea in a vaccine group be unexpectedly high (≥15%) and the rate in placebo recipients be 2%, with 100 postplacebo follow-ups and 50 postvaccine follow-ups for each vaccine dose and vaccine group, we would have an ∼86% power to detect a difference between vaccine and placebo (two-sided *z* test, alpha = 0.05).

## Supplementary Material

Supplemental material
