# Supplementary material for: Randomized, Placebo-Controlled, Double-Blind Phase 2 Trial Comparing the Reactogenicity and Immunogenicity of a Single Standard Dose to Those of a High Dose of CVD 103-HgR Live Attenuated Oral Cholera Vaccine, with Shanchol Inactivated Oral Vaccine as an Open-Label Immunologic Comparator
Source: Clin Vaccine Immunol. 2017 Dec 5;24(12):e00265-17. doi: 10.1128/CVI.00265-17 (PMC5717191; doi:10.1128/CVI.00265-17)
Supplement: Supplemental material [file supp_24_12_e00265-17__index.html]

Supplemental material 

# Randomized, Placebo-Controlled, Double-Blind Phase 2 Trial Comparing the Reactogenicity and Immunogenicity of a Single Standard Dose to Those of a High Dose of CVD 103-HgR Live Attenuated Oral Cholera Vaccine, with Shanchol Inactivated Oral Vaccine as an Open-Label Immunologic Comparator

## Supplemental material

- Supplemental file 1 -

  Table S1. Seroconversion rates, geometric mean titers, and geometric mean fold rises following oral vaccination with a single standard dose or high dose of CVD 103-HgR live oral vaccine or two doses of Shanchol inactivated cholera vaccine, using multiple imputation of missing values.

  PDF, 133K
