# Supplementary material for: Randomized, Placebo-Controlled, Double-Blind Phase 2 Trial Comparing the Reactogenicity and Immunogenicity of a Single Standard Dose to Those of a High Dose of CVD 103-HgR Live Attenuated Oral Cholera Vaccine, with Shanchol Inactivated Oral Vaccine as an Open-Label Immunologic Comparator
Source: Clin Vaccine Immunol. 2017 Dec 5;24(12):e00265-17. doi: 10.1128/CVI.00265-17 (PMC5717191; doi:10.1128/CVI.00265-17)
Supplement: Supplemental material [file CVI.00265-17_zcd012175546s1.pdf]

SUPPLEMENTAL TABLE 1. Seroconversion rates, geometric mean titers, and geometric mean fold-rises following oral vaccination with a single standard-dose (≥2x10^8 cfu) or high-dose (≥2x10^9 cfu) of CVD 103-HgR live oral vaccine or two doses (14 days apart) of Shanchol inactivated cholera vaccine, using multiple imputation of missing values. Also shown are effect size comparisons between the different vaccines at different study days.

Days after vaccination	Seroconversion rate (≥ 4-fold rise over baseline, percent)				Geometric mean titer (95% confidence interval)				Geometric mean fold-rise (GMFR, 95% confidence interval)			
	CVD 103-HgR		Shanchol		CVD 103-HgR		Shanchol		CVD 103-HgR		Shanchol	
	10^8 cfu	10^9 cfu	Post-1st dose	Post-2nd dose	10^8 cfu	10^9 cfu	Post-1st dose	Post-2nd dose	10^8 cfu	10^9 cfu	Post-1st dose	Post-2nd dose
**0**	-	-	-	-	28[a]<br>(21, 39)	28[b]<br>(21, 38)	47[c]<br>(32, 69)	-	-	-	-	-
**7**	53.6[d]<br>(38.4,68.7)	61.8[e]<br>(47.1, 76.5)	39.8[f]<br>(25.5, 54.1)		118[g]<br>(56, 248)	208[h]<br>(99,438)	113[i]<br>(67,191)	-	4.1[aa]<br>(2.0, 8·6)	7.4[bb]<br>(3.6, 15.4)	2.4[cc]<br>(1.4, 4.0)	-
**14**	68.9[j]<br>(55.2,82.6)	83·2[k]<br>(72.4,94.1)	56·0[l]<br>(41.9,70.1)		101[m]<br>(48, 216)	221[n]<br>(104, 471)	128[o]<br>(75, 218)	-	3·6[dd]<br>(1.6, 7.8)	7·9[ee]<br>(3.6, 17.4)	2·7[ff]<br>(1.6, 4.7)	-
**21**	75.4[p]<br>(62.6,88.2)	86.6[q]<br>(76.4,96.7)	-	68.5[r]<br>(55.2,81.8)	83[s]<br>(41,171)	179[t]<br>(87,369)	-	140[u]<br>(84,232)	2.9[gg]<br>(1.4, 6.2)	6.4[hh]<br>(3.0, 13.5)	-	3.0[ii]<br>(1.7, 5.0)
**28**	75.8[v]<br>(62.9,88.6)	87.0[w]<br>(77.0,96.9)	-	71.4[x]<br>(58.2,84.6)	71[y]<br>(38, 132)	162[z]<br>(87,301)	-	151[α]<br>(98,234)	2.5[jj]<br>(1.4, 4.6)	5.8[kk]<br>(3.1, 10.6)	-	3.2[ll]<br>(2.1, 4.9)
	[d] vs [e], p=0.43; [d] vs [f], p=0.19; **[e] vs [f], p=0.033**;[j] vs [k], p=0.10; [j] vs [l], p=0.19; **[k] vs [l], p=0.003**; [p] vs [q], p=0.17; [p] vs [r], p=0.45; **[q] vs [r], p=0.033**; [v] vs [w], p=0.17 [v] vs [x], p=0.63; [w] vs [x], p=0.060;				[a] vs [b], p=0.95 **[a] vs [c], p=0.045**; **[b] vs [c], p=0.034**; **[b] vs [c], p=0.034**; [g] vs [h], p=0.14; [g] vs [i], p=0.92; [h] vs [i], p=0.11; **[m] vs [n], p=0.046;** [m] vs [o], p=0.54; [n] vs [o], p=0.16; **[s] vs [t], p=0.042;** [s] vs [u], p=0.16; [t] vs [u], p=0.50; **[y] vs [z], p=0.010; [y] vs [α], p=0.020**; [z] vs [α], p=0.84				[aa] vs [bb], p=0.12; [aa] vs[cc], p=0.15; [bb] vs [cc], **p=0.003;** [dd] vs [ee], p=0.053; [dd] vs [ff], p=0.50; [ee] vs [ff], **p=0.009** [gg] vs [hh], **p=0.045**; [gg] vs [ii], p=0.98; [hh] vs [ii], **p=0.045** [jj] vs [kk], **p=0.008**; [jj] vs [ll], p=0.44; [kk] vs [ll], p=0.065			
**Effect size at day 7 after vaccination (2-sided 95% CI)**	Difference in seroconversion rates:<br>[e] vs [d] : 8.3 (-12.5, 29.0)<br>[d] vs [f] : 13.8 (-6.8, 34.3)<br>[e] vs [f] : 22.0 (1.8, 42.3)				Ratio of GMTs:<br>[h] vs [g] : 1.77 (0.71, 4.39)<br>[g] vs [i] : 1.04 (0.50, 2.18)<br>[h] vs [i] : 1.84 (0.88, 3.86)				Ratio of GMFRs:<br>[bb] vs [aa] : 1.8 (0.7, 4.4)<br>[aa] vs [cc] : 1.7 (0.8, 3.6)<br>[bb] vs [cc] : 3.1 (1.5, 6.4)			
**Effect size at day 14 after vaccination (2-sided 95% CI)**	Difference in seroconversion rates:<br>[k] vs [j] : 14.4 (-2.9, 31.6)<br>[j] vs [l] : 12.9 (-6.6, 32.3)<br>[k] vs [l] : 27.2 (9.7, 44.8)				Ratio of GMTs:<br>[n] vs [m] : 2.19 (0.87, 5.52)<br>[m] vs [o] : 0.79 (0.37, 1.68)<br>.[n] vs [o] : 1.73 (0.81, 3.68)				Ratio of GMFRs:<br>[ee] vs [dd] : 2.2 (0.8, 5.9)<br>[dd] vs [ff] : 1.3 (0.6, 2.9)<br>[ee] vs [ff] : 2.9 (1·3, 6·4)			
**Effect size at day 28 after vaccination (2-sided 95% CI**	Difference in seroconversion rates:<br>[w] vs [v] : 11.2 (-4.8, 27.2)<br>[v] vs [x] : 4.4 (-13.8, 22.6)<br>[w] vs [x] : 15.6 (-0.6, 31.9)				Ratio of GMTs:<br>[z] vs [y] : 2.27 (1.06, 4.85)<br>[y] vs [α] : 0.47 (0.25, 0.88)<br>[z] vs [α] : 1.07 (0.58, 1.99)				Ratio of GMFRs:<br>[kk] vs [jj] : 2.3 (1.1, 4.9)<br>[jj] vs [ll] : 0.8 (0.4, 1.4)<br>[kk] vs [ll] : 1.8 (1.0, 3.3)			
